# Visceral artery aneurysmal lesion of the omentum – a rare cause of spontaneous fatal intra-abdominal hemorrhage

**DOI:** 10.1007/s12024-022-00486-1

**Published:** 2022-06-01

**Authors:** Claire M. McClintock, Roger W Byard, Ruben Sebben, Neil E. I. Langlois

**Affiliations:** 1 Adelaide Medical School, Faculty of Health and Medical Sciences, University of Adelaide, SA Adelaide, Australia; 2 Forensic Science, Adelaide, SA Australia; 3 Queen Elizabeth Hospital, Woodville South, SA Australia

**Keywords:** Hematoperitoneum, Visceral artery, Aneurysm, Pseudoaneurysm, Unexpected death, Postmortem

## Abstract

We report unexpected death of a 72-year-old man due to a hemoperitoneum (1.9 L of blood in the abdominal cavity). Postmortem examination revealed that the cause of the hemorrhage was an arterial aneurysmal lesion in the greater omentum. The lesion measured 4 × 4 × 6 cm with a generally smooth wall, but with a focal area of rupture within a hemorrhagic region measuring 1 × 2 cm. There was a substantial feeding artery. Histological examination revealed features in keeping with a pseudoaneurysm, but also with some features of a true aneurysm. There was no history of trauma and the rupture of the aneurysmal lesion that had caused the hematoperitoneum was considered to be spontaneous. Prior to his death the deceased had attended hospital for epigastric pain, which was attributed to dyspepsia, but otherwise he had not had symptoms prior to his death.

## Case Report

A 72-year-old man was found deceased on his bed at his home address. He had a past history of prostatic carcinoma with metastases to the pelvis, in addition to ischemic heart disease, hypertension, chronic kidney disease, and atrial fibrillation. Four months prior to his death he had attended hospital for epigastric pain and was thought to have dyspepsia not related to peptic ulcer disease.

**Fig. 1:**
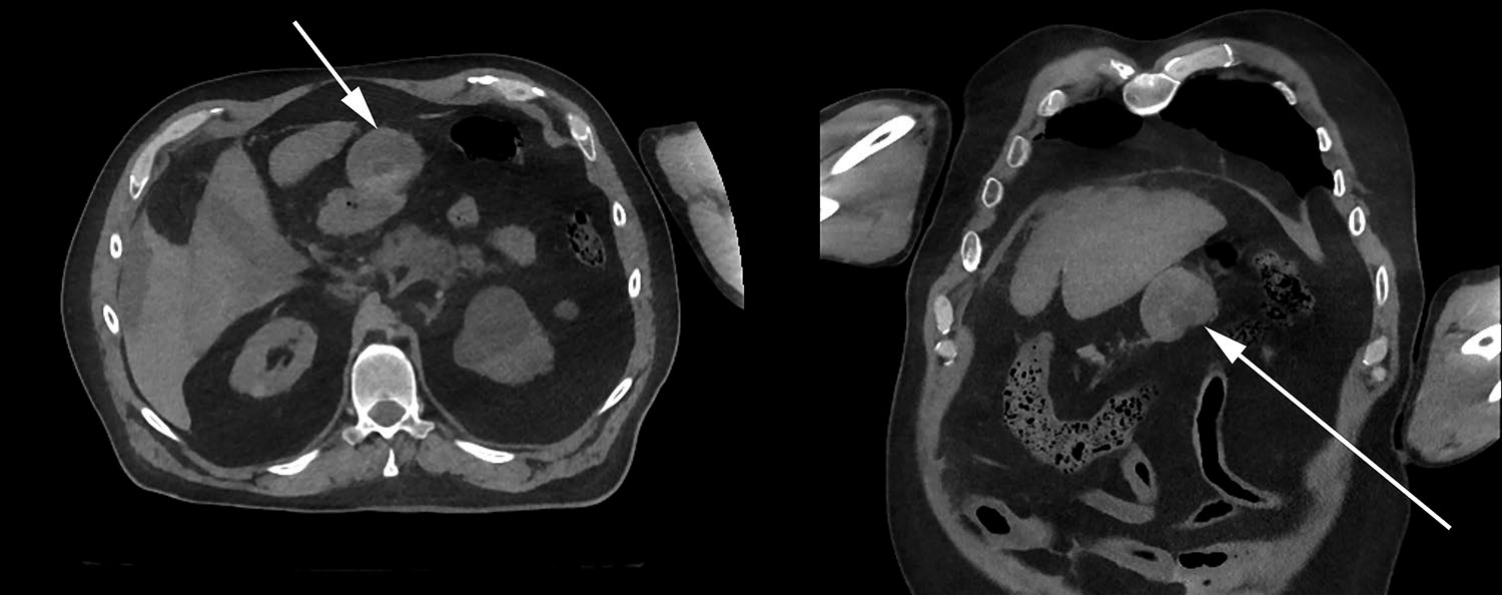
Postmortem CT (left axial slice, right coronial slice) showing visceral aneurysmal lesion (white arrows) and evidence of blood in abdominal cavity

A postmortem CT scan was performed which revealed fluid, in keeping with blood, around the liver, with similar fluid in the region of the spleen and in the pelvis. A poorly defined mass could be identified in the anterior upper quadrant of the abdominal cavity (Fig. 1). Postmortem CT angiography was not available.

At autopsy the deceased’s weight was 102 kg his height was 178 cm (BMI = 32). Opening the abdomen revealed 1.9 L of fluid and clotted blood predominantly in the right upper quadrant of the abdomen. In the greater omentum there was a fluctuant dark purple mass measuring 4 × 4 × 6 cm, with a generally smooth wall, which appeared to have ruptured on the right side within a hemorrhagic area measuring 1 × 2 cm. The mass was attached to the omentum, but had no other apparent connection to intra-abdominal structures. The contents of the abdomen and pelvis were otherwise normal with no alternative site of hemorrhage identified; the liver was not cirrhotic.

**Fig. 2:**
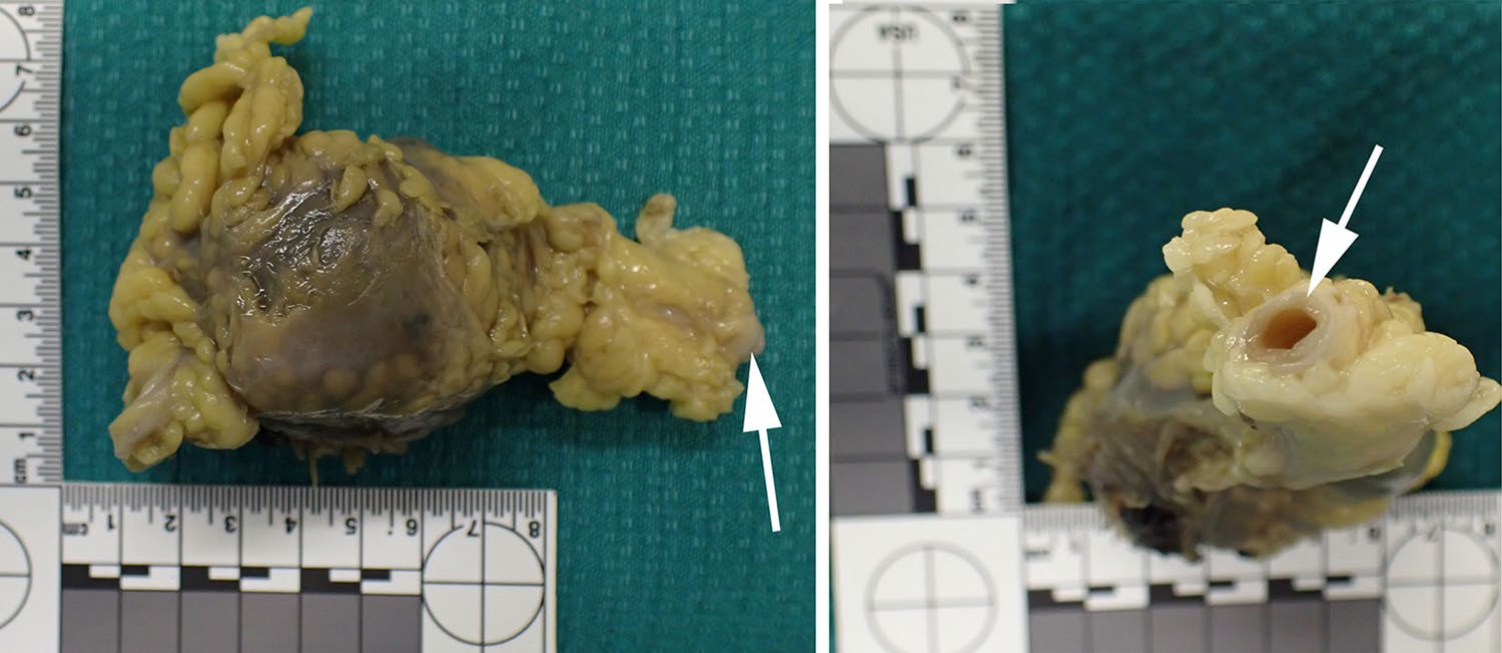
Macroscopic photograph of fixed specimen showing arterial feeding vessel (white arrows)

**Fig. 3:**
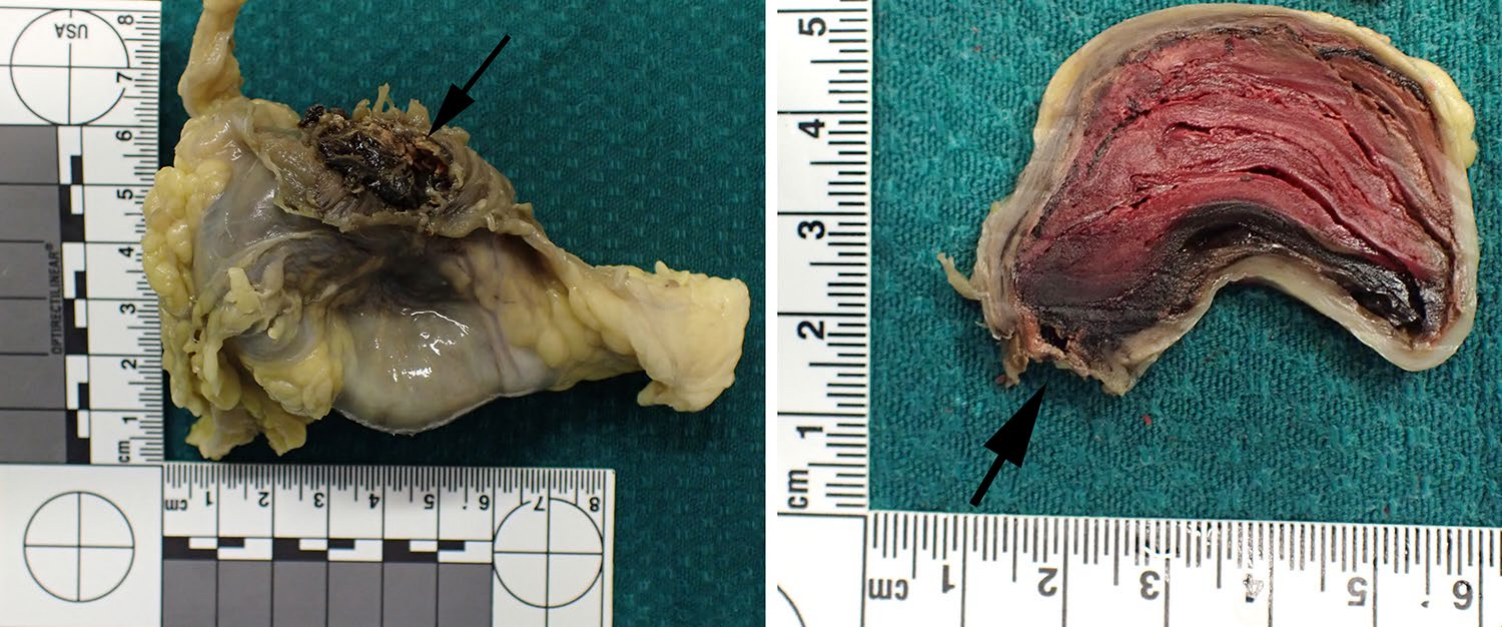
Macroscopic photograph of fixed specimen. Left - showing outer wall within omental fat and rupture site (black arrow). Right - section through fixed specimen at level of rupture site (black arrow) showing aneurysm contains laminated thrombus with apparently fibrous wall with patchy outer omental fat

Examination of the formalin-fixed specimen revealed a neck that comprised an artery measuring approximately 0.6 cm in diameter. This was in continuity with an approximately spherical, but partly collapsed, mass with an outer diameter of approximately 6 cm (Fig. 2). An exiting vessel was not identified. However, there was a dark, hemorrhagic area with yellow fibrinous material around an area of rupture of the wall. Sectioning revealed a fibrous white wall (approximately 0.1 cm thick) surrounding laminated thrombus (Fig. 3).

**Fig. 4:**
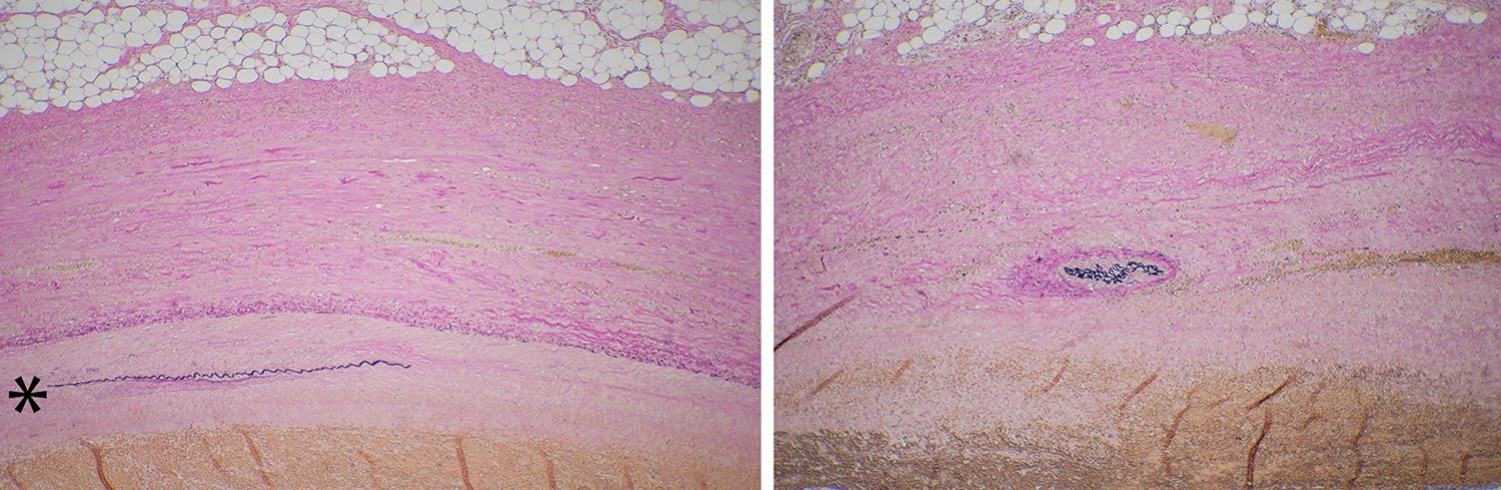
Photomicrograph of Elastic van Gieson stained section through wall from inner (bottom of image) to outer (top of image) of aneurysmal lesion. Left - wall comprises collagenous tissue with layer resembling internal elastic lamina (small arrows) apparently separating collagen from layer resembling reduplicated internal elastic lamina including layer of elastic tissue (marked with *); Right - apparent arterial vessel within wall (indicated by large arrow)

Histological examination of sections from the neck confirmed the presence of an arterial vessel with internal and external elastic laminae entering the mass. The artery showed variable loss of the internal elastic lamina with irregular areas of fibrosis and intimal thickening. The wall of the cystic mass was fibrotic with focal areas of lining resembling internal lamina; elsewhere there were mural arterial vessels (Fig. 4). In areas of the capsule there was patchy acute and chronic inflammation with hemosiderin deposition and reactive fibroblast proliferation.

Death was attributed to intra-abdominal hemorrhage from a visceral aneurysm/pseudoaneurysm. There were no other organic diseases which to have contributed to death and there was no evidence of trauma.

## Discussion

The initial finding in this case was the presence of blood in the abdomen, both on imaging and at autopsy. Due to a lack of history of trauma, the hemoperitoneum was attributed to rupture of a visceral aneurysm/pseudoaneurysm; however, there are a multitude of other possibilities to consider in the case of a hemoperitoneum.

Previously referred to as ‘abdominal apoplexy,’ idiopathic spontaneous intraperitoneal hemorrhage [1] is a rare, but often fatal condition [2]. The most common cause of intra-abdominal hemorrhage is, however, blunt trauma to a solid organ, such as the liver or spleen [3]. As there was no evidence of trauma in this case, non-traumatic causes are more likely including: central or visceral aneurysmal rupture [1], a complication of liver cirrhosis [4], hepatic or renal malignancy [1], inflammatory erosive processes including pancreatitis, [1] and iatrogenic [3]. In women, gynecological conditions including ruptured ectopic pregnancy [2] should be considered. Nonetheless, a proportion of cases with spontaneous hemoperitoneum do not yield a specific etiology [5].

In this case, the source of the fatal intra-abdominal hemorrhage was identified as a ruptured visceral aneurysmal lesion located in the greater omentum. The apparent origin from a large feeding vessel (Fig. 2) suggested that the lesion was most likely an aneurysm. However, the histological appearance of the aneurysmal lesion was mixed with areas suggesting an aneurysm from a dilated vessel, but with evidence of an arterial vessel in its wall and an incomplete elastic layer favouring a pseudoaneurysm (Fig. 4). A true aneurysm forms with all three arterial wall layers intact; whereas a pseudoaneurysm lacks a complete arterial wall [6].

The deceased had no history of abdominal trauma, previous surgery or pathology (such as infective endocarditis or sepsis) [7] prior to his death that could have predisposed to the formation of a visceral aneurysmal lesion. While the histological appearances of the feeder vessel with defects in the internal elastic lamina with replacement by fibrous tissue raised the possibility of fibromuscular dysplasia, [7] but there was no evidence of this elsewhere.

Visceral artery aneurysmal lesions include vascular aneurysmal lesions (aneurysms and pseudoaneurysms) of the splenic, hepatic, superior mesenteric, celiac, posterior duodenal, gastroduodenal, gastric and renal arteries [6]. Rupture of a visceral artery aneurysmal lesion carries a mortality of approximately 25% [8]. Such lesions are, however, rare, with a prevalence of 0.01 − 2% in the general population [9], although more are discovered as an incidental finding during radiological examinations [10] and the true prevalence may be closer to 0.78% [8]. Of the possible origins of visceral artery aneurysmal lesions, the omental visceral artery (gastroepiploic artery branch) aneurysm is considered to be particularly rare [11, 12].

Visceral artery aneurysmal lesions have been found over a wide age range (22–85 years) with a median age of 54 years and a male preponderance of around 3:2 [13]. This case occurred in a 72-year-old male. Spontaneous rupture is considered a risk if a lesion exceeds 2 cm in diameter [9] (the lesion in this case measured 6 cm in greatest dimension). Clinically, prior to rupture, there may be a history of abdominal pain [14] and it is noted that in this reported case the deceased had presented to hospital with upper abdominal pain that was diagnosed as non-ulcer dyspepsia (although his pain may have been unrelated to the lesion). If rupture occurs patients may present with pain and/or hypovolemic shock [13]; symptoms of bleeding (including melena and hematemesis) may be present if rupture has occurred into the gastrointestinal tract [15]. As in the current case, the first place of recognition and diagnosis may be at autopsy.

It has been noted that incidental visceral artery aneurysmal lesions may be encountered during clinical angiographic investigations [6, 8]. The increasing use of postmortem CT angiography may, therefore, raise the prominence of these lesions to pathologists, who could be otherwise unaware of the existence of visceral artery aneurysm as a cause of fatal intra-abdominal hemorrhage. If postmortem CT identifies the presence of hematoperitoneum without an apparent origin, postmortem CT angiography may considered as the next step in the investigative process to assess for the rare possibility of a visceral aneurysmal lesion of the omentum as a source of the haemorrhage.

## Key points

1. Visceral artery aneurysmal lesions (aneurysms and pseudoaneurysms) are a rare cause of hematoperitoneum
2. We describe a fatal spontaneous hematoperitoneum due to rupture of a visceral artery aneurysmal lesion in the greater omentum
3. Hematoperitoneum from a visceral artery aneurysmal may be diagnosed by CT scan with contrast angiography

## Data Availability

Data stored at Forensic Science SA. Access available to approved requests.
